# The evolution and distribution of noxious species of scorpions (Arachnida: Scorpiones)

**DOI:** 10.1186/s40409-017-0138-3

**Published:** 2018-01-04

**Authors:** Wilson R. Lourenço

**Affiliations:** Muséum national d’Histoire naturelle, Sorbonne Universités, Institut de Systématique, Evolution, Biodiversité (ISYEB), UMR7205-CNRS, MNHN, UPMC, EPHE, CP 53, 57 rue Cuvier, 75005 Paris, France

**Keywords:** Scorpion, Noxious species, Patterns of distribution, Biased results, Buthidae

## Abstract

This contribution attempts to bring some general information on the evolution and, in particular, on the geographic distribution of scorpion species noxious to humans. Since 95% of the scorpions incidents are generated by specimens of the family Buthidae C. L. Koch, the analysis will be limited to this familial group. As in previous similar contributions, the content of this work is mostly addressed to non-specialists whose research embraces scorpions in several fields such as venom toxins and public health. Only in recent years, efforts have been made to create better links between ‘academic scorpion experts’ and other academic non-specialists who use scorpions in their research. Even if a larger progress can yet be expected from such exchanges, crossed information proved to be useful in most fields of scorpion studies. Since the taxonomy of scorpions is complex, misidentifications and even more serious errors concerning scorpion classification/identification are often present in the general literature. Consequently, a precise knowledge of the distribution patterns presented by many scorpion groups and, in particular, those of infamous species, proves to be a key point in the interpretation of final results, leading to a better treatment of the problems caused by infamous scorpion species.

## Background

**Fig. 1:**
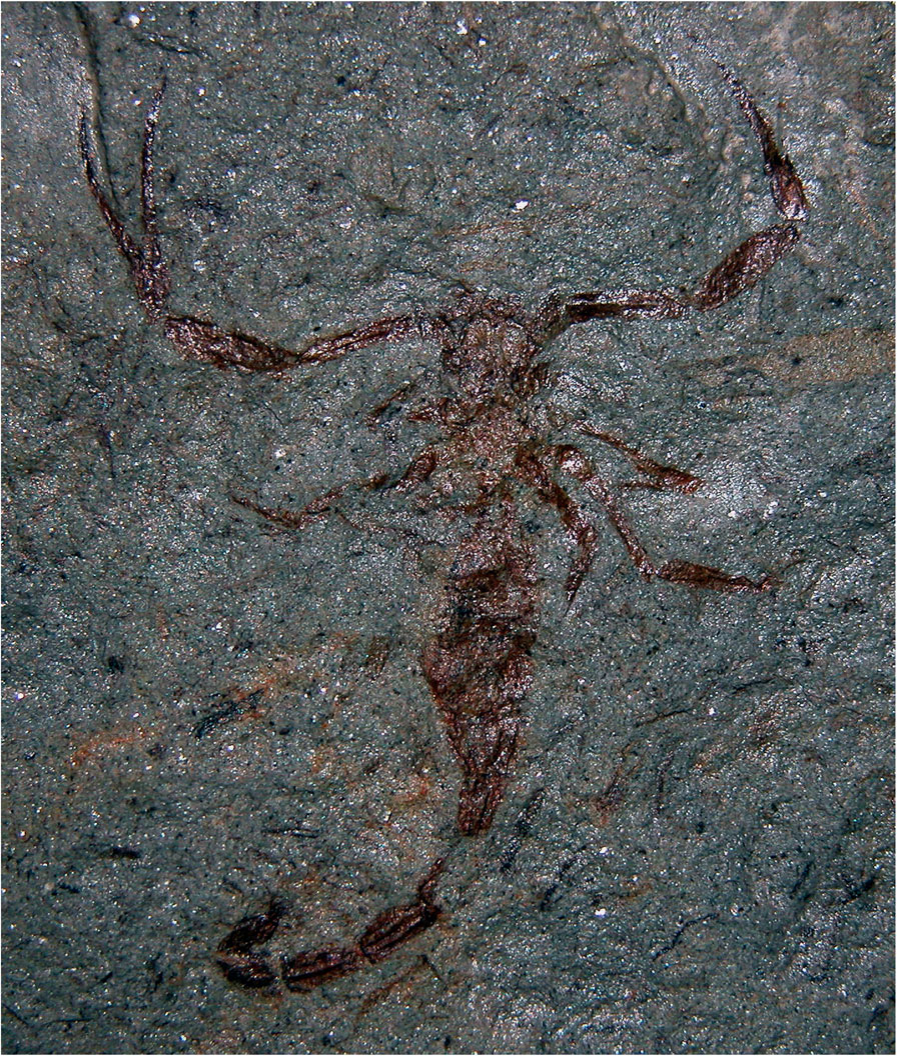
*Protobuthus elegans* Lourenço & Gall. Fossil scorpion from Early Triassic, France. The specimen, an adult male, clearly presents a telson

For many years now, there is a general consensus about the fact that scorpions can be classified among the most ancient and conservative arthropods both in origin and body morphology. They first appeared as aquatic organisms during the Silurian (approximately 450 million years ago – MYA) and apparently experienced few morphological changes since that period [1–3]. Because of their apparent conservative form some authors attempted to define the group as ‘living fossils’ (Fig. 1). However, this assumption is incorrect since scorpions most certainly underwent major biochemical, physiological, behavioral and ecological adaptations that have combined to ensure their continued success over the past 450 million years [2].

**Fig. 2:**
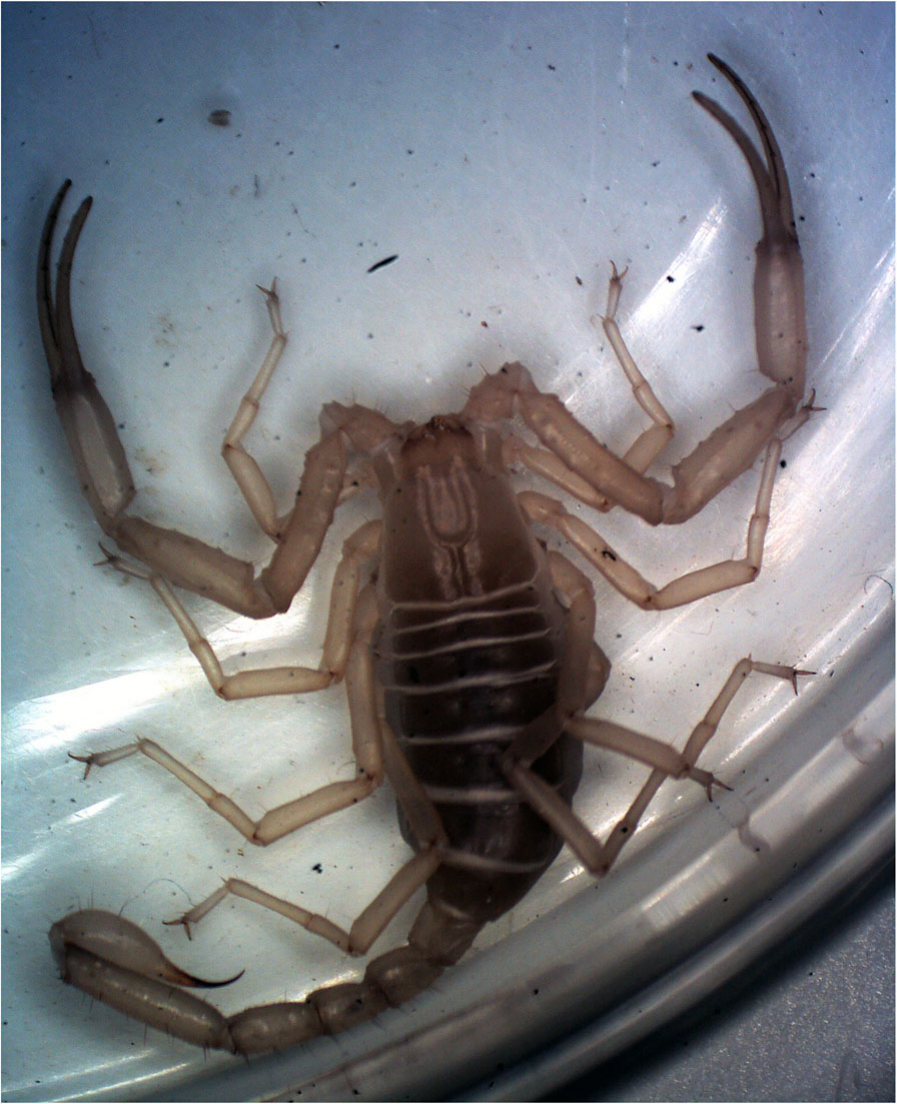
*Vietbocap thienduongensis* Lourenço & Pham (Pseudochactidae). A cave scorpion from Vietnam, the specimen is an adult male (copyright by D.-S. Pham, reproduced with permission)

Another widespread opinion is that scorpions are a rather depauperate group within the class Arachnida with approximately 2200 known species up to the present. It is obvious that the order can be considered modest when compared to that of other arthropods. The known number of species is much less impressive than those known for groups such as insects with over one million species or spiders with near 40,000 species. Nevertheless, the number of known scorpion species until the end of the nineteenth century (years 1899–1900) was approximately of 250. By 1975, this number reached 700 species. In present the days, only 40 years later, this number was multiplied by three. The increased number of described species is mainly due to the new techniques used in the prospection of scorpion in the field, but also to a better resolution in the definition of several populations [4–7]. The use of new techniques is often coupled with the exploration of new distinct habitats and microhabitats such as caves and organic soils. This leads to the discovery of completely unexpected elements (Fig. 2) [4, 8, 9]. In account of the progress achieved in the knowledge of different scorpion fauna such as those of tropical America, tropical Asia, Africa, Madagascar and Nearctic and Palearctic regions, one can expect that the total number of species may reach to 5000 or even more in the coming decades.

If the total of species surpasses now 2200, only a minority of these can be perceived by people in general. This is directly associated with the fact that most scorpion populations in nature are represented by very inconspicuous numbers of individuals, sometimes only a few thousands to a few hundreds. In other words, most scorpions are extremely rare.

Contrarily to scorpion experts who may consider these as fascinating animals for research, the attraction shown by humans in general is mainly connected with their negative reputation of ‘man killers’. Naturally, the species that represent a cause for fear and threat to humans are among those presenting dense populations represented by millions of individuals. The association between their noxious condition and massive populations, that can be found in pre-urban and even urban habitats, contribute to their infamous reputation [10, 11]. Nevertheless, only a limited number of species, probably less than 50, are actually responsible for serious or lethal incidents. From the beginning of the twentieth century on [12], it was obvious that the interest on scorpion research in many distinct biological fields was generated by the fact that a few species possess venoms with potent toxins, capable of killing humans [10, 11].

Two questions are often addressed concerning the noxious species of scorpions: (i) why a relatively small number of species possess venoms with potent toxins capable of killing humans? and (ii) why infamous species of scorpions have their distribution limited to certain regions of the world such as North and South America, North Africa, Middle East and certain regions of Asia? In other terms, why so many other regions of the world are spared from this phenomenon?

Scorpion venoms have been studied for more than a century, and some interesting results were revealed since then [13, 14]. Nevertheless, the *evolutionary significance* of mammal-specific toxins remains largely unsolved even if some attempts have been made to explain some possible evolutionary paths.

The deadliest species of scorpions belong to the family Buthidae C. L. Koch, with a few exceptions constituted by species of two other families, Hemiscorpiidae Pocock and Scorpionidae Latreille. In a didactical purpose, however, I will restrict my present analysis to elements of the family Buthidae. The conclusions I present in this work should be considered tentative, since our global knowledge of scorpion evolution presents yet numerous gaps. The main purpose is to bring some clarification, *even if partially*, to the previously noted questions, and to address possible explanations to non-specialists whose research embraces scorpions in several fields such as venom toxins and public health.

### The evolution of toxins

Although toxins may be one of the most important aspects to be considered in this analysis, I am only a zoologist; therefore, the subject is quite far from my domain of expertise. Consequently, I will limit myself to some general aspects.

For many decades, there have been an impressive amount of contributions to the domain of toxins – impossible to cite them all here – and quite many were synthesized in books [15]. Complementarily, some most interesting comparative analysis have also been produced more recently [13]. Nevertheless, in the majority of these studies, the basic questions were addressed on *how* these toxins act, and how they interact with a given organism once inoculated. Studies addressing the question on *why* a given toxin evolved to be active on a given organism are much less frequent. Only a few years ago a more precise hypothesis was formulated attempting to explain the causes of the evolution of some scorpion toxins, noticeable in relation to mammals [16].

The origin of mammal-specific toxins was suggested as an important issue in scorpion evolution. Old World lineages of Buthidae with very potent neurotoxic venom, such as the genera *Androctonus* Ehrenberg and *Leiurus* Ehrenberg, share separate mammal- and insect-specialized neurotoxins that are specific for *Na +* channels [17]. Inversely, New World genera such as *Centruroides* Marx and *Tityus* C. L. Koch have potent toxins acting on both mammals and insects. It was suggested as quite possible that the separate mammal-specific *Na +* toxins could have evolved during the aridification of the Palearctic region during the Tertiary period, when one of the most important selective factors was rapid radiation of small burrowing mammals (mostly rodents) in arid landscapes. Such newcomers to the scorpion environment, including rodents, would be a direct competitor for space (burrows) and in addition important nocturnal predators, as many of them are today [18]. This pressure could tentatively explain the emergence of specific mammal-targeting toxins, used mainly for defense, but not for foraging [17].

This hypothesis, although interesting, does not address all possible questions, since it cannot clearly justify the evolution of mammal-targeting toxins in New-World lineages and, in particular, in those present in regions where intensive aridification did not take place, such as in South America. Possibly the absence of more up to date explanations leads some recent authors to reconfirm this hypothesis [19].

**Fig. 3:**
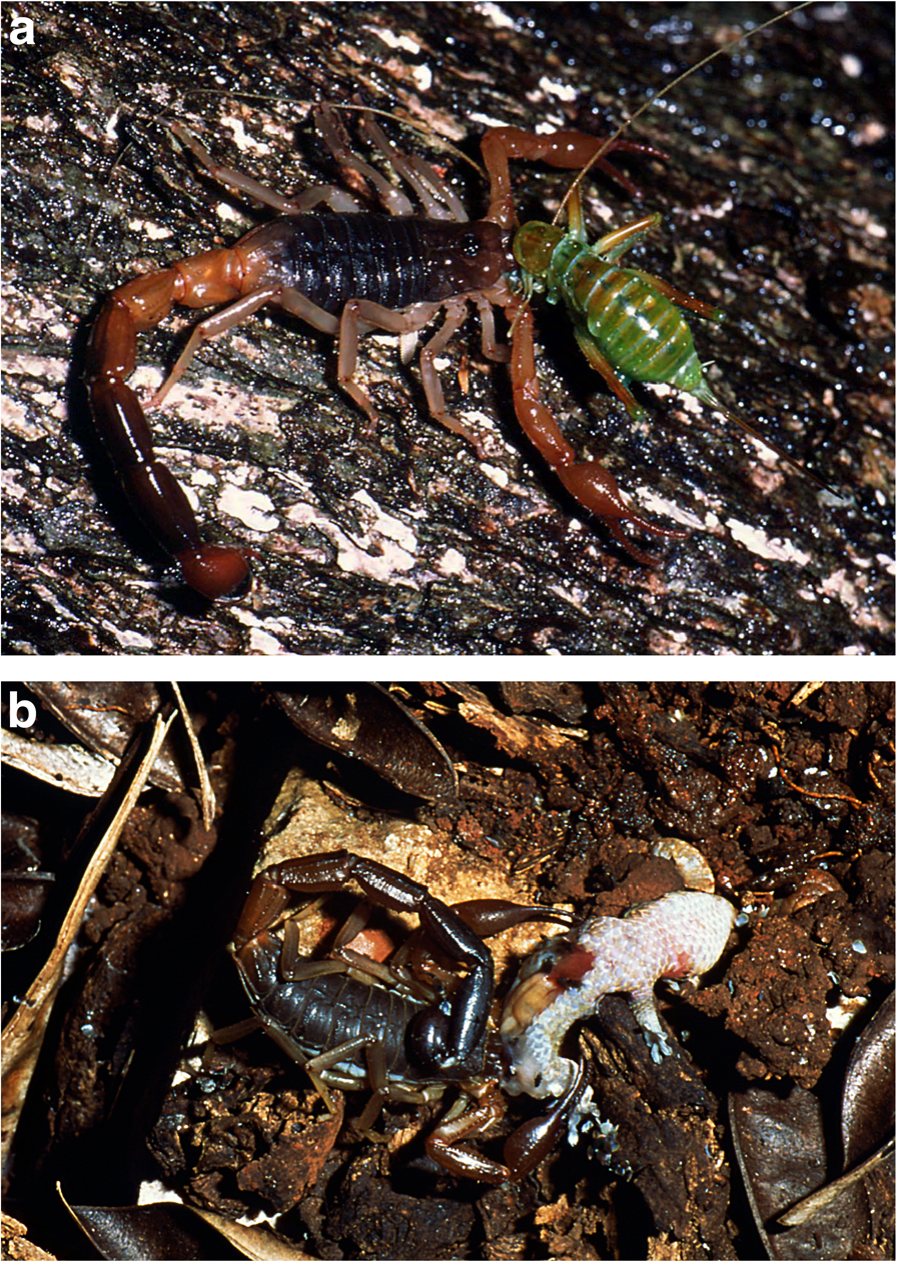
*Grosphus flavopiceus* Kraepelin from Madagascar. An adult female feeding respectively on (**a**) an insect (Orthoptera) and on (**b**) a gecko (Reptilia)

**Fig. 4:**
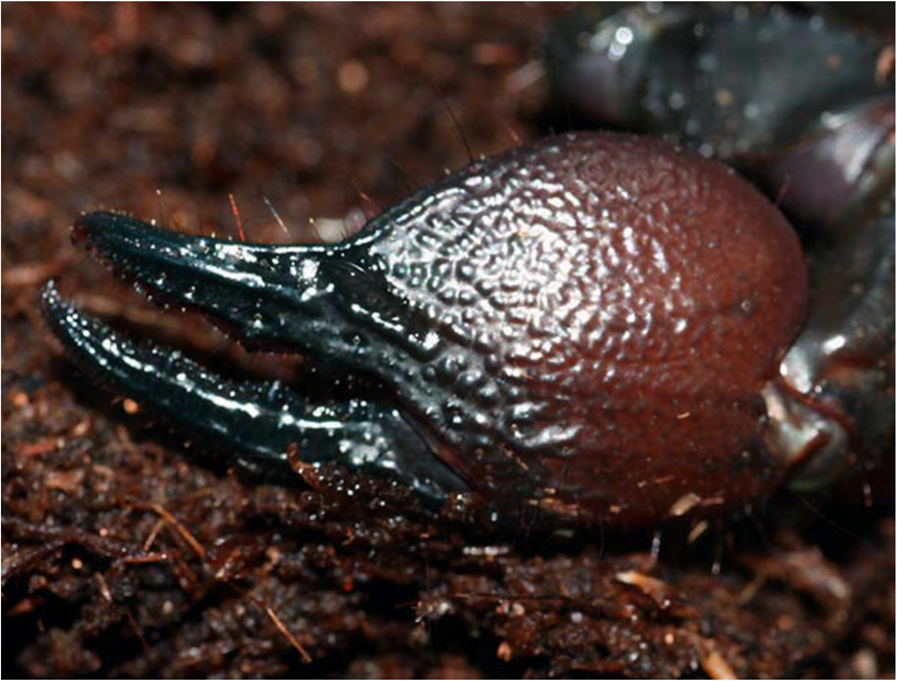
The very strong pedipalp-chela of a *Pandinus imperator* from Africa (copyright by E. Ythier, reproduced with permission)

Back to the more basic functions of scorpion venom and toxins, it is currently accepted that their primary function is associated with predation rather than defense [13]. Among modern (extant) scorpions, only a few species prey and feed on mammals (Fig. 3) and they do not belong to the family Buthidae. Only large scorpions generally can prey on small rodents, and most belong to the family Scorpionidae, genera *Pandinus* Thorell and *Heterometrus* Ehrenberg, for example. These large scorpions, some of which can reach almost 25 cm in length, possess very strong pedipalps (Fig. 4) and predation can be performed only mechanically, without the use of venom. In other words, they do not sting the prey.

Among Buthidae species, predation is largely concentrated on arthropods, mainly insects and spiders, and only isolated cases of predation on small vertebrates have been recorded [20]. Consequently, the evolution of very active mammal-specific toxins in several groups of the Buthidae family remains controversial.

### What fossils can tell us about the evolution of venoms?

As it was recently summarized, scorpions first appeared as aquatic organisms [2, 3]. In their evolutionary history, they almost certainly evolved from the Eurypterida (‘water scorpions’) since both groups share several common morphological features. Marine and amphibious scorpions most certainly persisted well into the Carboniferous (359–299 MYA) and some species probably reached the Permian (299–251 MYA) and Triassic (251–200 MYA) periods [21, 22]. The first unequivocally terrestrial (air-breathing) scorpion most certainly appeared on land during the late Devonian (416–359 MYA) or early Carboniferous [1, 23].

These early scorpions, almost all aquatic or amphibious, diversified rapidly into an impressive number of superfamilies and families. All these non-terrestrial fossil scorpions have been placed in one suborder the Branchioscorpionina Kjellesvig-Waering. Fossil scorpions, clearly accepted as terrestrial forms, are classified into a distinct suborder Neoscorpionina Thorell & Lindström, together with extant families. The suborder Branchioscorpionina includes 18 to 21 superfamilies and 41 to 47 families according to different authors [24, 25]. These numerous lineages are a clear indication of their early and great success. Moreover, because the fossil record is rather fragmentary, these more than 20 superfamilies are probably only a fraction of the total number that actually existed [1, 24]. It is clear, however, that only a few – possibly only one of these lineages – survived and evolved into present day species. Naturally, all extant scorpions live now in land.

The important number of fossil scorpion families accepted by strict paleontologists creates a divergence of opinion with neontologists. This divergence of opinion clearly indicates a taxonomic problem, and the difficulties of this type are often the result of different approaches in the studies performed by paleontologists and neontologists. The first working from the higher categories down and the second from lower categories up [24].

One important question concerns the age of extant scorpion lineages. Until recently, modern scorpion lineages were estimated to have been present since the very early Cenozoic [24]. This estimation was based on a few fossil records available for the Cenozoic and Mesozoic periods. Very recent discoveries for both the Cenozoic and Mesozoic periods based on both sedimentary and amber fossils attested that some extant lineages or at least proto-elements of these lineages are most certainly much older and were already present in the Lower Cretaceous [3, 26–29].

**Fig. 5:**
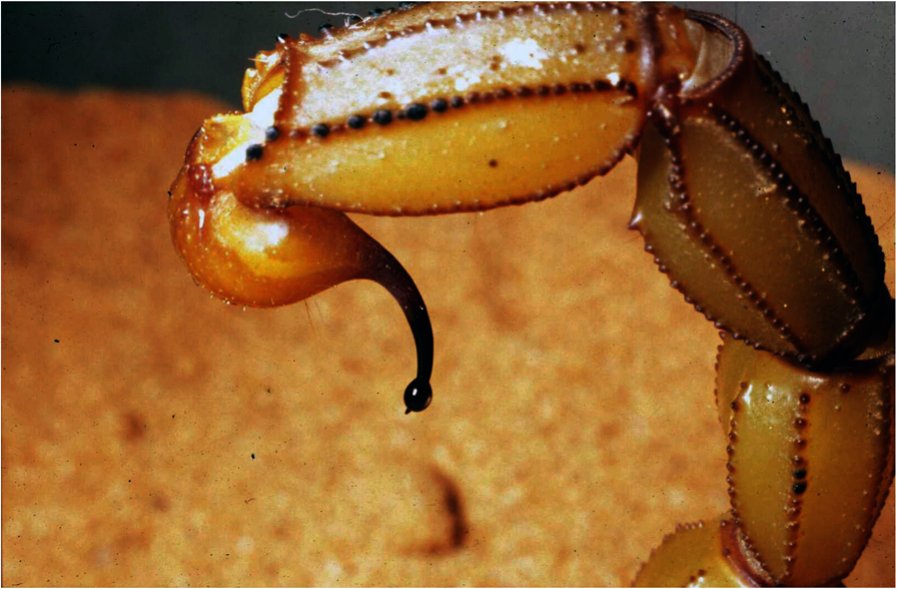
Telson of an extant buthid, *Buthus lienhardi* Lourenço. An adult female from Morocco

**Fig. 6:**
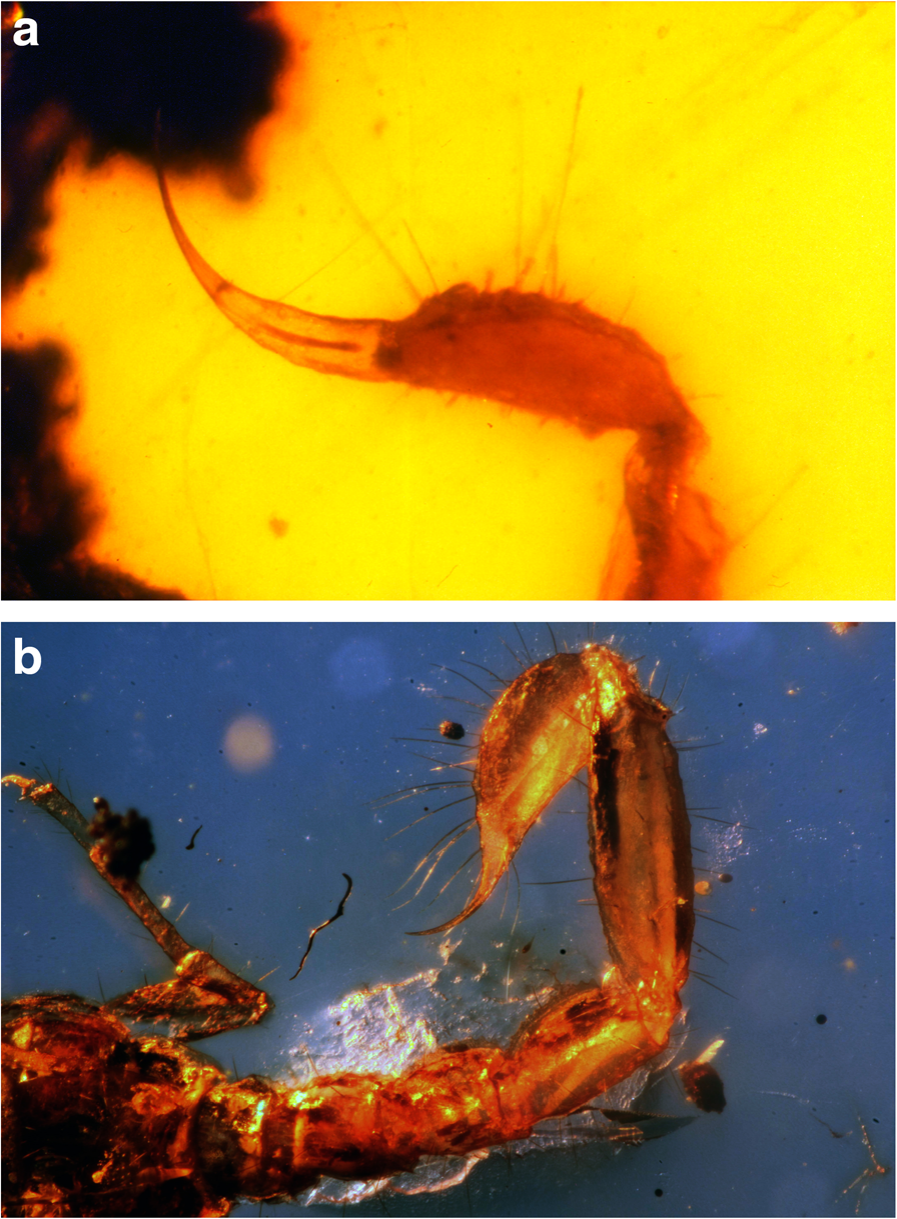
Telsons of two Early Cretaceous amber fossils from Burma, Myanmar. **a** *Palaeoburmesebuthus grimaldi* Lourenço (Palaeoburmesebuthidae). **b** *Chaerilobuthus schwarzi* Lourenço (Chaerilobuthidae) (**b** copyright by J. Velten, reproduced with permission)

Without any exception, all the extant scorpion species possess venom glands (Fig. 5). The presence of a telson with an aculeus and, in some cases, possibly tegumentary glands are also evident in several scorpion fossils from the Palaeozoic, Mesozoic and Cenozoic [1, 3, 23, 29–31] (Fig. 6).

Tegumentary glands are common in many arthropods and these probably evolved from the secretion of basic enzymes to more and more elaborate toxins, thus becoming complex venom glands. Based on the assumption that venom glands in scorpions have originally a predatory and digestive role, it is possible to suggest a process of coevolution between mechanical pattern of predation and the venomous function. This hypothesis appears as a good model for the elements of the buthoid lineage that generally have slender and/or weak pedipalps.

The precise evolution of the telson remains unclear. The structure was already present in Eurypterids and is yet common in several arthropod groups such as Xiphosura (horseshoe crabs). This posterior-most division of the body of an arthropod is not, however, considered as a true segment since it does not arises in the embryo from teloblast areas as do real segments. As for its possible original function in scorpions, the following paths can be suggested. The telson probably played a major mechanical role in predation, with the aculeus acting as a ‘spear-head’. Several fossil scorpions of the buthoid lineage show quite long aculei and this is also the case of a few extant genera of buthids such as the genus *Buthacus* Birula or *Buthiscus* Birula [3, 32]. Over the course of evolutionary time, tegumentary glands evolved into the telson vesicle, nevertheless, their primitive role was basically only digestive. In contrast, several non-buthid groups developed mechanical techniques of predation with the development of very strong and well-armed pedipalps. These groups do possess venom glands; however, the use of venom (toxins) for the capture of prey remains rather facultative.

Naturally, this previous argumentation, although of some interest, does not explains why some groups of extant buthids do possess very active venoms, in particular against mammals, while others do not. I will try to reconsider this point in the coming sections related to the evolution and distribution of noxious buthids.

### Buthoid composition and the evolution of distinct lineages

The classification and the phylogeny of scorpions are globally complex and cannot be detailed in this limited analysis. The buthoids probably appear as the most complicated group since they represent about 50% of all known scorpions and are the only group to be distributed in all the biogeographic regions of the earth.

Although some authors (mostly theoretical phylogeneticists) try to insist on the possible ‘monophyletic’ character of this group of scorpions, it seems that this cannot be the case. This superfamily, that most certainly comprises a small number of distinct families, cannot represent a homogeneous unit. Instead, it seems to be represented by four to five different evolutionary gradients [2, 33]. Nevertheless, the acceptation of several families within the buthoids or several subfamilies within the buthids is purely a theoretical exercise and has minor consequences on the present global approach to the evolution of noxious species.

Among the limited number of buthoid species possessing venoms formed by complex mixtures of highly specific toxins almost all belong to genera that can be placed in a high or even very high evolutionary level within the familial lineage. Coincidently, these genera including *Androctonus*, *Buthus* Leach, *Leiurus*, *Mesobuthus* Vachon, *Parabuthus* Pocock, *Centruroides* and *Tityus* have all been the subject of intensive biochemical and molecular research [17]. Most biochemical studies are concentrated on these groups because they are responsible for most scorpion incidents, but also because they are represented by conspicuous populations. Contrarily, almost no study has ever been performed on the most primitive lineages, both because these do not represent any threat to humans and because these scorpions are generally rare. These groups correspond to a number of relictual genera among which can be cited *Ananteris* Thorell, *Anomalobuthus* Kraepelin, *Akentrobuthus* Lamoral, *Birulatus* Vachon, *Egyptobuthus* Lourenço, *Himalayotityobuthus* Lourenço, *Lychasiodes* Vachon, *Microtityus* Kjellesvig*-*Waering, *Pseudouroplectes* Lourenço, *Sabinebuthus* Lourenço, *Tityobuthus* Pocock etc. I will try to associate these different lineages or gradients with fossils and biogeography in the following sections; but before some clarifications on biogeography patterns may be necessary.

### Biogeographic patterns presented by scorpions

Previously to considerations on the precise patterns of distribution of scorpions and in particular of the noxious species, it seems important to comment on some more general patterns defined in the last two or three decades.

Studies on scorpion biogeography are not recent. Attempts to interpret the observed models of distribution started since the end of the 19th century [34–36], but the general patterns of distribution then proposed were not in agreement. In fact, the viewpoints of the different authors were frequently quite distinct. These early general contributions have been followed only by regional biogeographic studies with a weak impact [37]. Only by the middle of the 1980s some new contributions on Neotropical scorpions allowed the definition of some biogeographic patterns [37–39]. The definition of these patterns became possible due to a better knowledge of the phylogeny of several groups, the application of recent hypothesis concerning climatic vicissitudes in tropical biomes during the late Cenozoic and Pleistocene periods and a much better knowledge of scorpion life-history strategies. Until the 1980s, no author ever took into consideration any parameters of life-history strategies to explain scorpion distribution. However, again starting in the 1980s, several biological and ecological studies demonstrated that most scorpions should be defined as equilibrium species, presenting therefore very predictable patterns of distribution [40, 41].

**Fig. 7:**
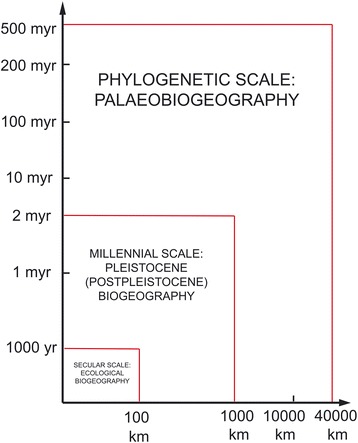
Division of biogeography into the three spatial-temporal scales of Udvardy (modified from Udvardy [42])

Subsequently, a more detailed biogeographical model was proposed [37] based on Udvardy’s [42] division of biogeography into three spatial-temporal scales (Fig. 7). This approach proved to be clear and didactic and three major biogeographical events were suggested to explain most of the patterns of distribution observed among scorpions today.

The first scale globally defined as phylogenetic or paleobiogeographic encompasses the evolutionary time of all biota and is limited in space only by the size of the earth [42]. On this scale, only historical factors can be assumed to have taken place since, for almost all ecological conditions, data are largely or totally unknown. At this level, the evolutionary process of biogeography is, to a considerable extent, a tributary of continental drift and plate tectonics. This new view shook the foundations of many theories adopted by older paleontologists and biogeographers [42].

Continental drift was taken into consideration by few authors when they discussed aspects of regional biogeography. The contribution of Lamoral [43] on the suprageneric classification of recent scorpions, with discussion on their zoogeography, was an important attempt to explain some general patterns and several of his suggestions are generally acceptable. He probably overestimated the role of dispersion when affirming that two major factors have influenced speciation and distribution patterns. One is the fragmentation of Pangaea and Gondwanaland; the other is the movement of Laurasian elements to the north of Gondwanaland. This second factor should be reconsidered. The process of ‘active’ dispersion should rather be interpreted as being a more ‘passive’ process in the dispersal sense as defined by Haffer [44]. This point is globally supported by the poor vagility presented in modern species of scorpions. Nevertheless, the present disjunctive distributions of several families and genera of scorpions remain unexplained. The cases of the present disjunctive distribution of some scorpion groups should be regarded as the result of the previous distribution of proto-elements of families and genera, followed by a vicariant process. The precise mechanism of these processes is not, however, always known. In conclusion, the main event responsible for the distribution of scorpions on a paleogeographic scale is the fragmentation of Pangaea and subsequent continental drift. The difficulties to explain the discontinuous distribution of some familial and generic groups point not only to the great geological age of these groups, but also to the relict faunas and biogeographical patterns that they exhibit currently.

The second scale used in scorpion biogeography can be defined as millennial or Pleistocene biogeography. Between the development of the earth’s crust and the Pleistocene epoch several events took place, many of which were related to the continuous drift of the continents. Some examples are mountain building, differential erosion, epicontinental seas, climatic-vegetational fluctuations, changes of world sea level and the formation of major river systems. These events took place during the Cenozoic over a period of 60 MYA, and have influenced the present biogeographical patterns of scorpions. Climatic-vegetational fluctuation most certainly played a major role, starting on the late Cenozoic period, but having a major impact during Pleistocene time [44, 45].

During many years most contributions concerning tropical regions stated that the biogeographic and diversity patterns observed in these regions could be explained by the long stability of tropical forests over millions of years [46, 47]. Subsequent studies on geology, paleoclimates and palynology, especially in Amazonia and Africa [48–51], demonstrated that this presumed stability was a fallacy. In fact, although the temperatures in tropical lowlands remained ‘tropical’ during glacial periods (3 to 5 °C lower than today), the forest broke into isolated remnants during cool dry periods (glacial phases). The remnants of forest expanded and coalesced during warm humid periods (interglacial phases). Conversely, nonforest vegetation expanded during glacial and retreated during interglacial phases (as at present). Data from geoscience, however, have been insufficient to indicate the precise areas of changing forests and nonforests and, in particular, the areas in which forests remained during arid phases, presumably serving as refugia for animal and plant populations. Nevertheless, in the Neotropical region, studies on the biogeographical patterns of scorpions [38, 52, 53] suggested several endemic centers that are well correlated with the results obtained by Prance [48] on woody plants, and Haffer [54] on birds.

The third scale also used in scorpion biogeography is defined as ‘ecological biogeography’. However, this scale was globally rejected in pioneer biogeographic studies, mainly because it was biased towards two major considerations:

(i) an almost total lack of knowledge of life history strategies, more precise data on this subject was only available from the 1970s and 1980s on, but was almost the only preoccupation of ecologists;
(ii) a generalized opinion, even among modern biologists, that scorpions are capable of withstanding radical changes in environmental conditions, and therefore of being very good colonizers. This assumption is obviously fallacious. With growing knowledge on scorpion life history strategies it was clearly evident that many, if not most scorpions, are equilibrium species, which tend to inhabit stable and predictable natural environments, produce single egg clutches, do not store sperm, have long life spans, present low population densities, have a very low r_max_, show weak mobility, and are highly endemic [39–41].

In contrast, it is true that a minority of scorpions show traits of ‘opportunistic species’. Most of these opportunistic elements belong to the family Buthidae, but a few can also be associated with other families such as Euscorpiidae and Hormuridae. They are marked by ecological plasticity and are readily capable of invading disturbed environments. They may produce multiple clutches from a single insemination, have elaborate sperm storage capabilities [55], short embryonic development, short life spans, high population densities, rapid mobility, and are widely distributed. The study of these opportunistic species is globally of little interest for the definition of biogeographical models.

Opportunistic species thrive in disturbed and unpredictable environments that can be the result of natural causes (e.g.; volcanic activity) or are directly associated with human action. Several classical examples can be outlined, including the populations of the Neotropical species *Centruroides gracilis* (Latreille) that are established in the Canary Islands for almost two centuries [35, 41]. In addition, although the originally Sri-Lankan species *Isometrus maculatus* (DeGeer) has nowadays a worldwide distribution in tropical and semi-tropical regions, this species has been transported by humans during the last four centuries. The replacement of species is well illustrated in several islands of Eastern Asia where natural volcanic activity and human impact are important [56].

In continental regions, opportunistic species can rapidly occupy habitats disturbed by human activities, where the original native species have been selected against, thus leaving their ecological niches vacant. Several examples are known by scorpion experts, as in the case of numerous noxious species of the genus *Centruroides* distributed in Mexico, and largely influenced by the anthropic action. One particular example is well known by biologists in general and concerns the remarkable expansion of the noxious Brazilian species *Tityus serrulatus* Lutz & Mello during historical times [57, 58]. This particular case was already discussed in detail [10, 11].

### The association of different evolutionary lineages with fossil records

When the existence of 4 to 5 distinct evolutionary gradients was first suggested [33], the knowledge of well-preserved fossils from Mesozoic and Cenozoic periods was yet poor. In the last two decades, however, a good access to new fossil elements become possible bringing some new clarifications on the relationship between extant elements and those from early Mesozoic to late Cenozoic. A few conclusions are possible.

**Fig. 8:**
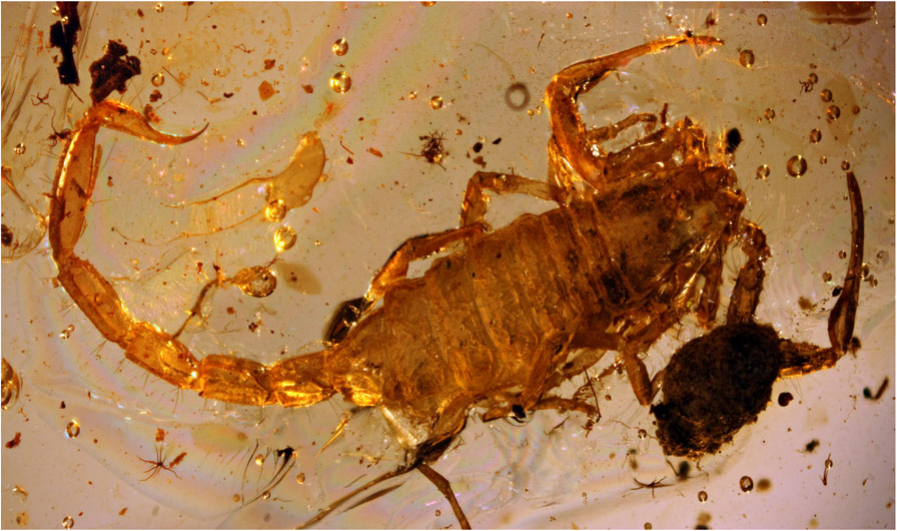
*Betaburmesebuthus bellus* Lourenço (Palaeoburmesebuthidae). Young male from Early-Cretaceous amber of Burma (copyright by C. Gröhn, reproduced with permission)

Some sedimentary fossils from the early Triassic such as the family Protobuthidae Lourenço & Gall can already be classified among elements of the buthoid in *sensu* *lato* [23]. However, no precise connections can be done to precise extant generic groups for instance. More recent Cretaceous amber fossils suggest some early links with extant lineages, and some well-defined families such as the Archaeobuthidae Lourenço from Cretaceous of Lebanon and Palaeoburmesebuthidae Lourenço from Cretaceous of Burma can clearly be assigned to the a buthoid lineage [3, 26, 27, 59]. However, the link between the most common Cretaceous burmite genera, *Palaeoburmesebuthus* Lourenço and *Betaburmesebuthus* Lourenço (Fig. 8), with extant genera remains vague. In fact, these two burmite elements show very primitive characters that apparently vanished in recent forms [3]. Nevertheless, in a few other isolated cases, elements from Cretaceous burmite were proven to be directly associated with the Buthidae family and to extant elements. One example is *Archaeoananteroides maderai* Lourenço that was clearly related to the extant genus *Ananteroides* Borelli [60].

**Fig. 9:**
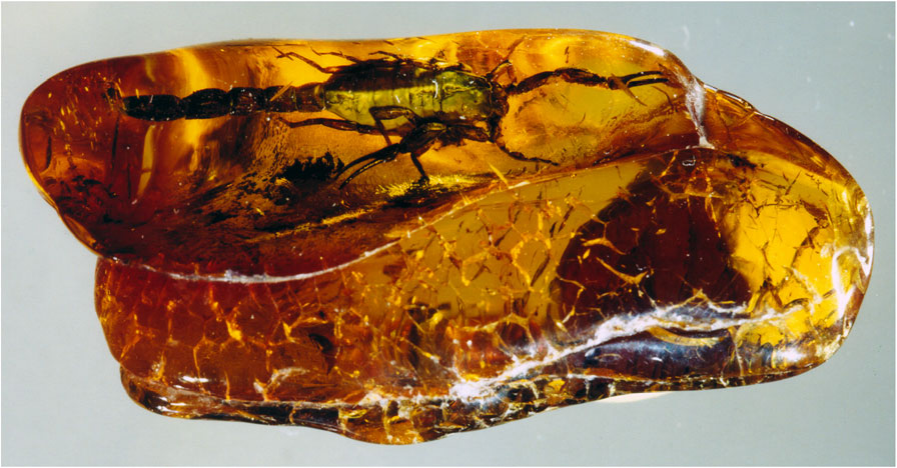
*Palaeoananteris ribnitiodamgartensis* Lourenço & Weitschat (Buthidae). Adult male from Early-Cenozoic Baltic amber

**Fig. 10:**
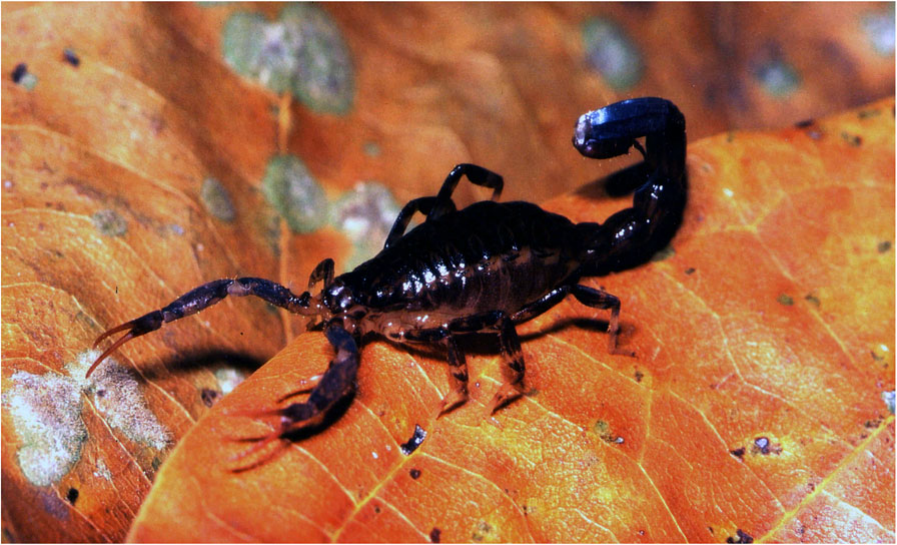
*Ananteris charlescorfieldi *Lourenço (Buthidae, Ananterinae). Adult female from Bolivia

Although Cenozoic sedimentary fossils are extremely rare [31], a number of amber elements from this period has been discovered and studied in the last three decades. Earlier elements from this period can be dated from the Palaeocene to Eocene and globally correspond to pieces found in Baltic amber [61]. All studied scorpions from this period were classified in the family Buthidae, and with one single exception, were all assigned to the subfamily Ananterinae Pocock (Fig. 9) [3, 30], which can be ranged among the lower evolutionary buthoid gradients [33]. It is important to recall that all the extant elements belonging to the Ananterinae are globally not noxious and although rare present a wide range of distribution over different continents such as Africa, tropical America, and Asia (Fig. 10). The present pattern of distribution of the Ananterinae suggests a panbiogeographic model and the group was most certainly dominant over all emerged lands in early Cenozoic.

A number of late Cenozoic elements are also known from Dominican and Mexican amber. The dating of this American amber can be controversial, but is normally suggested as Oligocene-Miocene. The characteristic trait of the elements found in this late Cenozoic amber is that all, without exception, can be classified among typical American extant groups such as *Centruroides*, *Tityus* and *Rhopalurus* Thorell. Only distinct species were described from this type of amber [62–64]. It is important to notice that these genera can be classified among the most evolved within the evolutionary gradients defined for the buthoids [33]. Unfortunately, no fossil records are available for other noxious groups such as the African/Middle East genera *Androctonus*, *Buthus*, *Hottentota* Birula, *Leiurus*, *Mesobuthus* and *Parabuthus*. Nevertheless, the fossil chronology suggests the evolution of noxious species, probably from the middle of Cenozoic epoch, and correlates well with the hypothesis suggesting that mammal-specific toxins would have evolved during aridification of the Palearctic region during the Tertiary period [16].

The evolution of these more evolved buthoid groups certainly took place in many regions of all emerged lands. Their present, and somewhat, more located regions of distribution can largely be attributed to more recent geological and paleoclimatical vicissitudes that took place from the middle to the end of the Cenozoic epoch and even during the more recent Pleistocene period. In all cases, these correspond well to the events defined for the second and third scales used in scorpion biogeography, defined as millennial/Pleistocene and ecological biogeography. In the next section, I will try to provide some examples.

### Noxious species in the Saharo-Sindian scorpion fauna

The Saharo-Sindian region comprises most of the faunal elements distributed from northwest Africa to India via the Middle East [65]. This naturally includes several genera containing noxious species such as *Androctonus*, *Buthus* and *Leiurus*. The present composition of this fauna is, in fact, the heritage of ancient faunas present in North Africa and the Palearctic region since the beginning of or, at least, middle Cenozoic times [66, 67]. North Africa and the Palearctic region have experienced numerous paleoclimatological vicissitudes during the last few million years, some even in more or less recent Quaternary periods. The Sahara, for example, has undergone a series of wet periods, the most recent occurring from 10,000 to 5000 years before present (BP), and it was not until about 3000 years BP that the Sahara assumed its present arid state [68]. Even though recent studies suggest that the Sahara desert may be much older than was previously thought [69], it seems reasonable to postulate that extremely arid areas have always existed as patchy desert enclaves, even when the general climate of North Africa enjoyed more mesic conditions.

In these arid and desert regions of both the North African Sahara and the Palearctic region, a specialized scorpion fauna would have evolved in response to the aridity. These ‘ancient lineages’ adapted to arid conditions, undoubtedly correspond to extant groups such as genera *Androctonus*, *Buthacus*, *Buthiscus*, *Buthus*, and *Leiurus* some of which are typically psammophilic. It is important to emphasize the fact that these lineages must have been present in North Africa for at least 10 to 15 MY [70, 71], and also in the Palearctic region during subsequent periods [67]. To notice that among the lineages positively selected, not all correspond to groups possessing noxious species.

In contrast, other lineages less well adapted to aridity and, previously, only present in more mesic environments, have regressed markedly in their distribution with the expansion of the desert. In consequence, they have, in some cases, already experienced negative selection and doubtless will eventually disappear. In other cases, populations have been reduced to very limited and patchy zones of distribution, sometimes with remarkable disjunctions in their patterns of distribution. It is noticeable to recall that these less well-adapted or more primitive lineages have no noxious species. One good example is the genus *Butheoloides* Hirst [72].

The models of the distribution of North African scorpions observed today can be summarized as follows: a core Saharian region, described by Vachon [66] as the ‘central compartment’, in which only the groups best adapted to xeric conditions (such as the genera *Androctonus*, *Buthacus*, *Buthiscus*, *Buthus*, and *Leiurus*) are distributed. In the peri-Saharian zone, surrounding most of the central compartment, some remarkable disjunctions occur. One of them is presented by the genus *Microbuthus* Kraepelin with species in Mauritania and Morocco in the West and other species in East Africa and Middle East [73]. Finally, several groups, sometimes less well adapted to xeric environments, have their populations limited to refugia [66]. These refugia are represented by the Saharan massifs, such as Hoggar, Aïr and Adrar, as well as other elevated regions in Mauritania and Occidental Sahara. Some of the endemic genera, such as *Cicileus* Vachon, *Lissothus* Vachon, *Egyptobuthus* and *Pseudolissothus* Lourenço provide useful examples [66, 74, 75]. A similar picture can be observed in several regions of the Middle East, in particular among the mountain ranges of Iran and Afghanistan [67].

### Species from the Amazon region

The biogeographical models observed for Neotropical scorpions suggested direct correlations with climatic-vegetational fluctuations during the Pleistocene. One of these models confirms the presence of polymorphic species in the Amazon region, mainly belonging to the genus *Tityus*. Some of these species are strongly noxious while others are not. A few can have wide ranges of distribution from French Guiana to Peru, but most are limited to zones in eastern or western Amazonia. It was suggested that during the dry periods of the paleoclimatic episodes when the forest was reduced to small patches, widespread species became fragmented into several isolated allopatric populations. These isolated populations of ecologically adaptable species (which is the case of some *Tityus* spp.) rapidly recolonized the reestablished forest during wet episodes. Previously isolated populations thereby became contiguous. Temporary reproductive isolation did not produce genetic barriers (at least for scorpions) and only minor morphological differences evolved. Where species reunited, the variation was no longer geographically correlated.

Another observed model corresponds to disjoint distributions of species of a given genus present in savanna and rainforest formations. Examples can be provided by species almost exclusively adapted to savannas (genus *Rhopalurus* Thorell) or to rainforests (genus *Hadrurochactas* Pocock). Isolated endemic populations provide good evidence for the hypothesis of past connections between the savannas of central Brazil and present enclaves in Amazonia and Guyana, since during past dry periods the savanna formations probably coalesced. The presence of enclaves of forest (regionally called *brejos*) inside arid formations of northeast Brazil equally suggest past connections between Amazonia and the Atlantic forest in Brazil. This hypothesis is supported by the biogeographical pattern in Amazonia presented by scorpions of the genus *Hadrurochactas*. It is obvious that the vicissitudes faced by the Amazon region did not act only on the noxious species, but globally those of the genus *Tityus* seem to be particularly exposed.

### Patterns of distribution of noxious species in southeastern Brazil and Mexico

The patterns of geographic distribution presented by noxious species in southeast Brazil and Mexico are largely associated with the human impact on the environment and several ecological factors associated with these species. In fact, if most scorpion species require predictable and stable environments, some species can strongly be opportunists. This is the case of members of the genera *Centruroides*, *Tityus* and *Isometrus* Ehrenberg that can exhibit marked ecological plasticity and be readily capable of invading disturbed environments. They produce multiple clutches from a single insemination, have elaborate sperm storage capabilities [55], short embryonic development, short life spans, high population densities, rapid mobility, and are widely distributed. These opportunistic species are of little use for establishing biogeographical patterns, but several are strongly noxious to humans. The invasion of habitats disturbed by human impact is well known in Brazil and Mexico, but also in other regions of the world. When this form of secondary succession is associated with noxious, opportunistic species, public health problems can arise. For more details, refer to Lourenço [10, 11].

### Early isolation of Madagascar

**Fig. 11:**
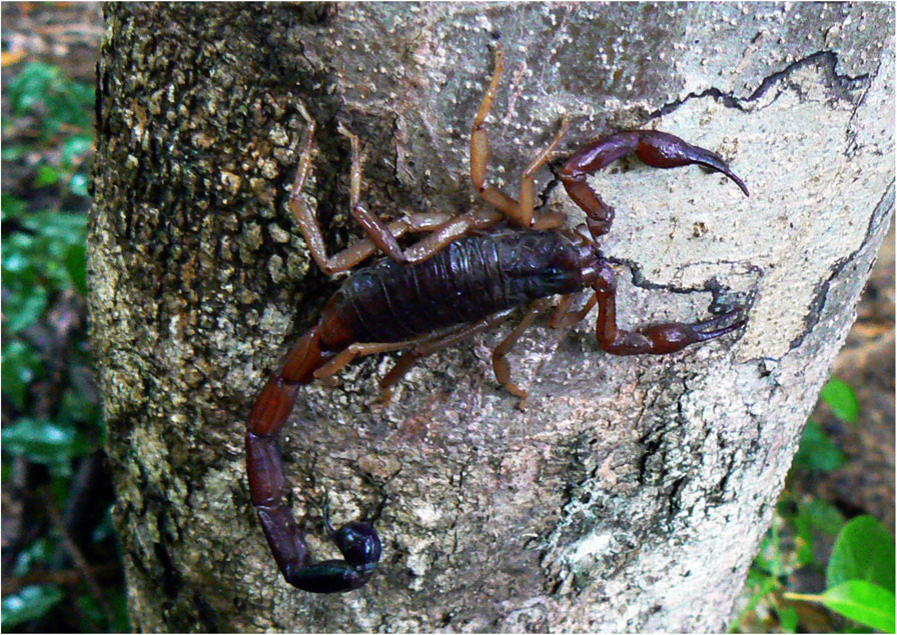
*Grosphus ankarana* Lourenço & Goodman (Buthidae). Female from the north of Madagascar

Some regions of the world such as the great island of Madagascar show a rather primitive fauna including that of scorpions [76]. In fact, geologically speaking, Madagascar become isolated from other continental masses during the Gondwana break and this isolation now persists for 80 to 110 MY [77]. The faunal influences from the nearby lands were reduced to a minimum and all scorpion lineages presented in the island today can be considered as primitive. This includes also all the buthoid elements, represented by the families Buthidae and Microcharmidae Lourenço. Some of these buthoid elements are among the most primitive known and some others can be classified as being in an average gradient of evolution [4, 78, 79]. Consequently, in spite of the significant number of species present in the island, no noxious species are known. Only a few isolated cases of moderately severe scorpion stings were reported for 2 or 3 species of the genus *Grosphus* Simon, with a particular attention to *Grosphus ankarana* Lourenço & Goodman, a large species (Fig. 11) distributed in the northern range of the island.

## Conclusions

As in my recent articles published in *JVATiTD* [10, 11], the main objective of this contribution is to bring some general and broad information about noxious species of scorpions which can have a medical importance. This note is addressed to non-specialists who use this group in their research. Once again, I attempt to demonstrate that scorpion diversity and patterns of distribution are much more complex than it seems at first sight. This is particularly true because the evolutionary history of the group is very old and most evolutionary patterns remain unknown.

As already emphasized previously [10, 11], since the group’s diversity is quite important, a similar view should be applied in what concerns the diversity of toxins. Presently, only a very limited number of species are used in the study of venoms and toxins, mainly because they represent a threat to humans. However, a better knowledge of scorpions in general by non-experts may encourage their interest to do new research using a broader array of scorpion lineages, in particular those that can bring more information on the evolution of venoms.

## Abbreviations

BP: before present
MYA: million years ago
